# Structural Changes on MRI Demonstrate Specific Cerebellar Involvement in SLE Patients—A VBM Study

**DOI:** 10.3390/brainsci11040510

**Published:** 2021-04-16

**Authors:** Johan Mårtensson, Theodor Rumetshofer, Jessika Nystedt, Jimmy Lätt, Petra Nilsson, Anders Bengtsson, Andreas Jönsen, Pia C. Sundgren

**Affiliations:** 1 Department of Clinical Sciences Lund, Logopedics, Phoniatrics and Audiology, Faculty of Medicine, Lund University, 221 00 Lund, Sweden; 2 Department of Clinical Sciences Lund, Diagnostic Radiology, Faculty of Medicine, Lund University, 221 00 Lund, Sweden; theodor.rumetshofer@med.lu.se (T.R.); jessika.nystedt@med.lu.se (J.N.); pia.sundgren@med.lu.se (P.C.S.); 3 Department of Clinical Sciences Lund, MR Physics, Faculty of Medicine, Lund University, 221 00 Lund, Sweden; jimmy.latt@med.lu.se; 4 Department of Clinical Sciences Lund, Neurology, Faculty of Medicine, Lund University, 221 00 Lund, Sweden; petra.nilsson@med.lu.se; 5 Department of Clinical Sciences Lund, Rheumatology, Faculty of Medicine, Lund University, 221 00 Lund, Sweden; anders.bengtsson@med.lu.se (A.B.); andreas.jonsen@med.lu.se (A.J.)

**Keywords:** lupus, neuroimaging, VBM, cerebellum

## Abstract

The purpose of this study is to investigate possible differences in brain structure, as measured by T1-weighted MRI, between patients with systemic lupus erythematosus (SLE) and healthy controls (HC), and whether any observed differences were in turn more severe in SLE patients with neuropsychiatric manifestations (NPSLE) than those without (non-NPSLE). Structural T1-weighted MRI was performed on 69 female SLE patients (mean age = 35.8 years, range = 18–51 years) and 24 age-matched female HC (mean age = 36.8 years, range = 23–52 years) in conjunction with neuropsychological assessment using the CNS Vital Signs test battery. T1-weighted images were preprocessed and analyzed by FSL-VBM. The results show that SLE patients had lower grey matter probability values than the control group in the VIIIa of the cerebellum bilaterally, a region that has previously been implied in sensorimotor processing in human and non-human primates. No structural differences for this region were found between NPSLE and non-NPSLE patients. VBM values from the VIIIa region showed a weak positive correlation with the psychomotor speed domain from CNS Vital Signs (*p* = 0.05, *r* = 0.21), which is in line with its presumed role as a sensorimotor processing area.

## 1. Introduction

Systemic Lupus Erythematosus (SLE) is a chronic autoimmune disease that follows a pattern of relapsing-remitting, meaning that patients typically experience periods of more severe respectively milder symptoms that alternate over time. SLE is about nine times more common in women than men [1] and the disease onset usually occurs in women of childbearing age [1,2,3]. Both the central and peripheral nervous system are commonly affected (21–95% of all patients; [4]) along with most organ systems which, by extension, risks negative effects for the individual’s psychosocial well-being [5,6,7,8,9,10,11].

Researchers and clinicians speak of neuropsychiatric SLE (NPSLE) in cases where SLE is accompanied by neurological and/or psychiatric symptoms and of non-neuropsychiatric SLE (non-NPSLE) for patients without those symptoms. Estimates of the proportion of SLE patients that suffer from neuropsychiatric symptoms (NP) range widely between 21 and 95% [4]. These symptoms can comprise several complaints, for example mild cognitive impairment or mood disorders as well as more severe instances, such as stroke, epilepsy, psychosis, or microangiopathy [12]. Cognitive impairment is common in SLE patients entailing possible adverse effects on perceived quality of life, even when symptoms are not severe enough to be clinically relevant [5,13]. Aiming to provide a unifying framework for the diagnosis of NPSLE, the American College of Rheumatology (ACR) defined a total of 19 NP syndromes, the presence of any of which can serve as a basis for an NPSLE diagnosis [12,14]. Due to the poorly understood underlying biology and the heterogeneous and diffuse phenotype of the disease, diagnosing and managing NPSLE poses a considerable challenge. The pathophysiology behind the disease is diverse and can include production of autoantibodies, intrathecal production of proinflammatory cytokines that affect the blood-brain barrier [15], cardiovascular disease [16], ischemia [17], thromboembolism [18], and atherosclerosis [19].

Aiming to differentiate between NP symptoms that are in fact caused by SLE, a number of attribution models have been proposed [20]. A widely cited, population-based study from Finland found that almost all of the screened SLE patients (91%) had experienced at least one NP event [5]. However, if mild instances (most prominently cognitive impairment, headache and mood disorders) were not taken into account, the prevalence of NPSLE in the sample dropped to 46%. A later meta-analysis corroborated these results similarly finding cognitive impairment, headache and mood disorder to be the three most common NP occurrences in NPSLE [12]. Factoring in these three syndromes, the overall prevalence of NPSLE amounted to 56%, although there was substantial variability in the prevalence estimates of the individual underlying studies [12]. There is some debate whether cognitive deficits are in fact a primary symptom of SLE itself or rather a secondary consequence of other manifestations of the disease, such as pain, fatigue or poor sleep quality [13]. Corticosteroids, a commonly used medication to treat SLE, have also been proposed as a trigger of cognitive impairment, though the available empirical evidence is inconclusive [11]. In a study of the effects of SLE on cognition in patients free from corticosteroid mediation, Nishimura and colleagues [11] found evidence of neurocognitive impairment in nearly 30% of SLE patients as opposed to 7% of healthy controls. The cognitive domains that stood out the most were immediate recall, complex attention, executive function, and psychomotor speed, with the latter being the most powerful differentiator between the two groups. The most commonly reported NP syndrome is neurocognitive impairment [11]. Despite of this, as of now our knowledge about the underlying causes of neurocognitive dysfunction in SLE is still very limited. However, alterations in white matter structure and the presence of antiphospholipid antibodies have been suggested as contributing factors [11].

Magnetic Resonance Imaging (MRI) is the most commonly used tool for evaluating suspected brain lesions in SLE patients and has proved useful in advancing our understanding of the neural correlates of the disease. White matter hyperintensities and atrophy are frequent findings in SLE patients, whilst at the same time it remains elusive what exactly characterizes the nature of the relation between white matter lesion load and symptom severity [6,21,22,23]. Earlier findings using MR spectroscopy [24,25], diffusion weighted (DWI) and diffusion tensor imaging (DTI) [26,27,28] have shown metabolic alterations as well as microstructural changes in both white and grey matter in SLE and NPSLE patients when compared to healthy controls [29]. Both grey matter and white matter volume in the cerebrum are known to decrease as an effect of SLE [30]. Findings on grey matter compared for example to DTI or MR Spectroscopy are still somewhat scarce. As of yet, there is no empirical evidence for aberrant cerebellar grey matter among SLE patients, whilst both cerebellar ataxia [31,32,33] and vasculopathy [34] have previously been observed in small samples of SLE patients.

Based on previous findings on structural brain alterations in SLE patients [35,36,37,38,39], we hypothesize that grey matter probability, as measured using Voxel-Based Morphometry (VBM), will be lower in SLE patients (indicating brain atrophy) as compared to HC, and that any observed differences will be more pronounced in NPSLE patients than non-NPSLE patients.

## 2. Results

**Figure 1:**
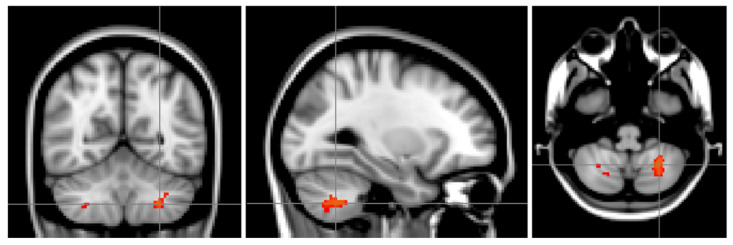
SLE patients had lower grey matter probability values in the left (and to a lesser extent the right) VIIIa area of the cerebellum when compared to controls. The images are oriented as seen from the front, so left above is right and vice versa. The images depict a study specific template brain with the effects between the groups (NeuroPsychiatric Systemic Lupus Erythematosus (NPSLE) and non-NPSLE versus controls) superimposed.

**Figure 2:**
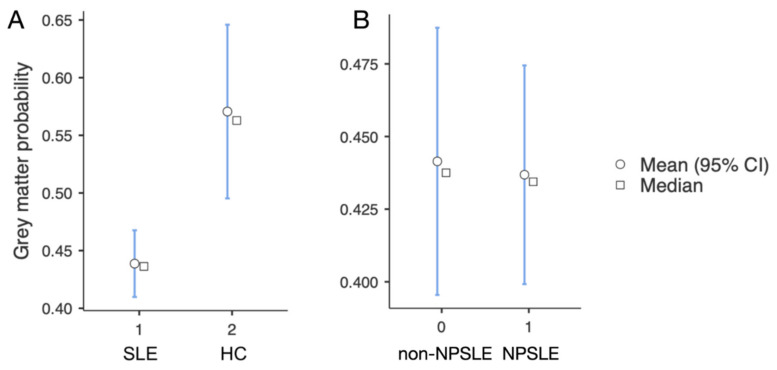
(**A**) SLE patients showed lower VBM values than HC in the left VIIIa. (**B**) No differences were found between the two groups of SLE patients (NPSLE (NeuroPsychiatric Systemic Lupus Erythematosus) and non-NPSLE) in this region. Values represent the mean values from the entire region depicted in Figure 1.

**Figure 3:**
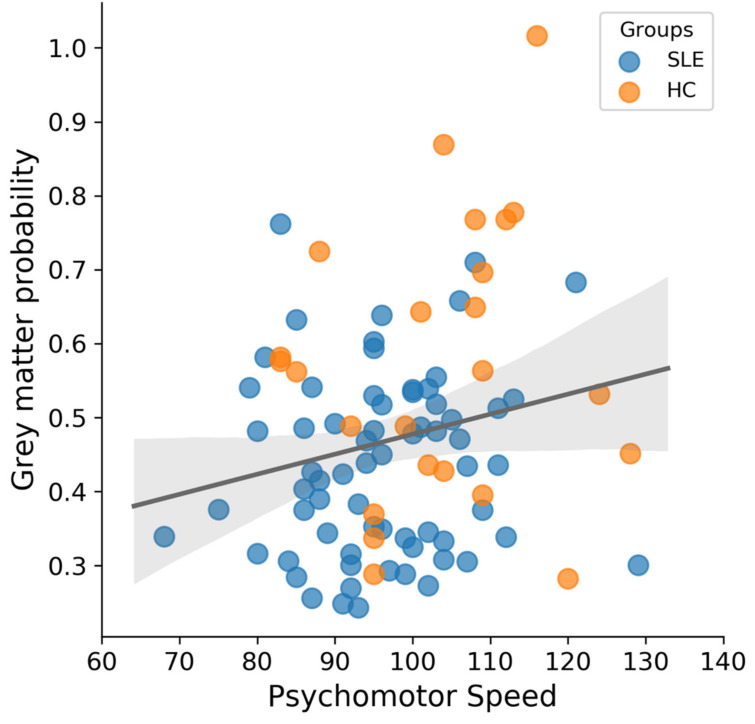
Extracted grey matter probability values from the region of interest depicted in Figure 1 A correlate weakly with psychomotor speed (*p* = 0.05, r = 0.21), suggesting that the VIIIa of the cerebellum is involved in motor activity. The image depicts patients with Systemic Lupus Erythematosus (SLE) and Healthy Controls (HC) in separate colors.

The SLE groups (non-NPSLE and NPSLE) were characterized by different morphometry than HC in the VIIIa area of the left cerebellum and to a much lesser extent in the left VIIa area. A similar pattern was visible in the right cerebellum (see Figure 1 and Table 1), with no observable effects elsewhere in the brain. Compared to the control group, SLE exhibited lower VBM (Voxel-based morphometry) values in this region (*t*(91) = −3.92, *p* < 0.001, see Figure 1). However, no difference was found for that same area between NPSLE and non-NPSLE (*t*(67) = 0.35, *p* = 0.728, see Figure 2). VBM values from the region of interest were compared to psychomotor performance, since the cerebellum has traditionally been implicated in motor functions. Additionally, psychomotor speed is one of the cognitive abilities that has previously been shown to be reduced in SLE patients [5,11,14]. A weak correlation was found between VBM values and test performances between SLE and HC (*p* = 0.05, r = 0.21), indicating that the VIIIa area might be relevant for psychomotor speed (see Figure 3).

## 3. Discussion

Whole brain VBM revealed lower grey matter probability in the VIIIa of the cerebellum (bilaterally, but mainly in the left hemisphere) in right-handed SLE patients when compared to HC. However, VBM values did not differ as a function of whether or not patients experienced NPSLE manifestations. VIIIa has previously been linked to the processing of information related to motor function in non-human primates [40] and most likely also in humans, where it is hypothesized to play a role in motor representations [41,42]. The VIIb in turn, has been linked to both pain and motor processing [43]. The presented grey matter structural differences in this area were associated with psychomotor speed performance. Aberrant cerebellar structure and function has earlier been reported in SLE patients [31,32,33,34,44,45]. However, to our knowledge this is the first time that, originating from a relatively sizeable cohort, cerebellar deviations were detected in this population by means of grey matter measures. Another merit of the present study is the fact that changes in brain structure could be linked to concrete cognitive outcomes. In order to lend further support to the current findings, additional research is needed on the suspected role of cerebellar dysfunction underlying some of the cognitive deficits that are typically observed in SLE patients. Ideally, this will involve a combination of different imaging and behavioral measures, as well as large and well-defined samples. Whilst not completely conclusive, the present study ties well into earlier findings of deviations in the cerebellum of (NP)SLE patients. Cerebellar pathologies have also previously been observed in patients with multiple sclerosis (MS), a chronic demyelinating inflammatory autoimmune disease that shares a number of defining features with SLE [46,47]. Several studies have reported the presence of lesions and atrophy in cerebellar white matter of MS patients [48,49,50], thereby providing additional evidence for the partial overlap of the clinical picture characterizing both SLE and MS. In one study, MS patients with cerebellar dysfunction were found to perform weaker than MS patients free from cerebellar dysfunction with respect to attention and verbal fluency. Nonetheless, no direct link between lesion load and behavioral deficits could be established. However, motor-related skills were not assessed in that study [50]. Overall, the current findings point towards cerebellar integrity as a potentially important in SLE, and the region should be kept in mind in future studies investigating the disease.

## 4. Conclusions

In conclusion, the present study points to a tentative link between deviations in brain structure and an associated behavioral outcome which brings us a step closer towards delineating the presumed role of the cerebellum in the aetiopathogenesis of SLE. Determining disease-specific brain abnormalities along with their behavioral consequences will be crucial not only for a better general understanding of the condition, but also for the gradual development of a more targeted and reliable scheme for the diagnosis and treatment of SLE.

## 5. Materials and Methods

Participants. This cross-sectional study included 69 female SLE patients (mean age = 35.8 y, range = 18–51 y) and 24 age-matched female healthy controls (HC) (mean age = 36.8 y, range = 23–52 y). The study was approved by the local ethics committee at Lund University, which follows the national guidelines set out by the Swedish Research Council. Written informed consent was obtained from all participants prior to data collection. Inclusion criteria applying to all prospective participants were female gender, age between 18 and 55 years and right-handedness (due to possible differences in the brain organization of left-handers which risk to systematically distort MRI findings). In addition, HC had to be free from any previously diagnosed autoimmune or NP condition. In order to be included in the SLE group, patients had a clinical diagnosis of SLE and fulfilled at least at least four of the American College of Rheumatology classification criteria for SLE [51]. All subjects also underwent self-assessment questionnaires: Fatigue Severity Score (FSS; [52]), Visual Analog Scale (VAS; [53]) and the Montgomery Asberg Depression Rating Self-Report (MADRS-S; [54]). All participants were evaluated by both, a rheumatologist and neurologist including assessment using the SLE-disease activity index 2000 (SLEDAI-2k; [55]), and the Systemic Lupus International Collaborating Clinics/American College of Rheumatology (SLICC/ACR) Damage Index (SDI; [56]). Participants with severe depression (MADRS-S > 34), claustrophobia (in consideration of the narrow tunnel in an MRI) and major intracerebral pathologies were excluded. Patients with known NP events defined by the rheumatologist to be associated with the patient’s diagnosis of SLE where classified as NPSLE patients, defined according to the ACR case definitions for NPSLE [57]. In total, 28 patients were classified with Non-NPSLE and 41 patients with NPSLE.

Neuropsychological evaluation. All participants underwent standardized computerized neurocognitive testing with an experienced neuropsychologist present who provided an introduction and additional assistance when required. The software package Central Nervous System Vital-Signs (CNS-VS) was deemed well-suited for studying SLE patients. It captures cognitive domains that are commonly affected in this patient population [14] and the subtests the battery contains overlap to a large extent with the one hour test- battery that the 1999 ACR committee proposed for use in cases of suspected NPSLE [57]. Based on the participants’ performance on seven subtests, it provides scores for nine basic cognitive functions: composite memory, verbal memory, visual memory, executive functioning, processing speed, psychomotor speed, reaction time, complex attention, and cognitive flexibility [58].

MRI. All MRI examinations were performed on a 3T MR Scanner (Siemens MAGNETOM Skyra, Erlangen, Germany) located at Skåne University Hospital. The imaging protocol included the following sequences: T2w-TSE, T2w-FLAIR, DTI, and 3D T1w-MPRAGE (1 mm isotropic, TE/TR/TI = 2.54/1900/900). MPRAGE was performed twice, i.e., before and after intravenous contrast administration of 0.2 mL/kg of Gadolinium-DOTA (Dotarem^®^, Gothia Medical, Guerbet. France). Only the non-Gadolinium T1w-MPRAGE images were used for the present analysis. [11].

**Figure 4:**
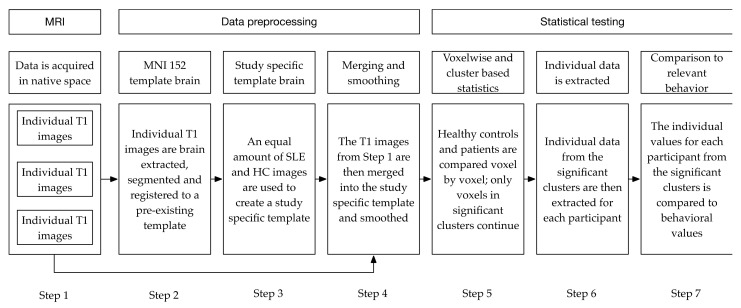
Schematic depicting the different data processing steps. The individual steps are described below in the sections MRI (Magnetic Resonance Imaging), Data preprocessing and Statistical testing.

Data preprocessing. To investigate whether cerebral and/or cerebellar grey matter structure differed between SLE patients (NPSLE and non-NPSLE) and HC, a Voxel-Based Morphometry (VBM) analysis based on whole brain T1w MRI data was conducted. VBM is an automated technique that compares values across voxels between groups, e.g., diseased populations or controls. It is typically used to infer differences in brain tissue, the presence of atrophy or other related changes in tissue as an effect of disease [59]. Structural data were preprocessed using a standard VBM protocol using FSL tools [60,61,62]; http://fsl.fmrib.ox.ac.uk/fsl/fslwiki/FSLVBM, accessed on 16 April 2021. Figure 4 shows a schematic overview of the different preprocessing steps. T1-weighted images were brain-extracted and grey matter-segmented before registering them to MNI 152 standard space using non-linear registration [63]. The resulting images were then averaged and flipped along the x-axis to create a left-right symmetric, study-specific grey matter template, which was based on an equal amount of SLE patients and controls. Next, all native grey matter images were non-linearly registered to the study-specific template. The resulting grey matter images were smoothed using an isotropic Gaussian kernel with a sigma of 2 mm.

**Statistical testing**. The smoothed images were then analyzed with voxelwise General Linealr Model (GLM) using permutation-based non-parametric testing (see Figure 4). This was fully correcting for multiple comparisons using Threshold-Free Cluster Enhancement (TFCE; [64]), which is a method for finding clusters in your data without having to define them earlier. Regions of interest, i.e., the resulting clusters, were then extracted using fslmaths and fslstats [61]. Fslmaths was used to mask the original image using the previous extracted regions of interest and fslstats to calculate the mean non-zero voxels for each participant within these areas. Finally, the resulting individual values were exported and correlated with relevant behavioral findings (CNS Vital Signs Psychomotor Speed) using Jamovi [65], R [66] and JASP [67].

## Figures and Tables

**Table 1:** Cluster index and size for Figure 1. Cluster index was extracted using the FSL tools Cluster and Atlasquery and are corrected for multiple comparisons.

			Location of Maximum Intensity Voxel (mm)			Location of the Centre of Gravity for the Cluster (mm)			
Cluster	Voxel Size	Maximum *p* Value	X	Y	Z	X	Y	Z	Localization
1	126	0.03	−26	−58	−46	−27.4	−60.1	−44.9	35% L. VIIIa, 5% L. VIIb
2	52	0.04	38	−48	−52	38.5	−50.8	−51.5	65% R. VIIIa, 30% R. VIIb, 4% R. Crus II
3	12	0.05	16	−66	−42	17.8	−65.7	−43	21% R. VIIIa, 15% R. VIIb, 1% R. Crus II
4	4	0.05	28	−60	−46	29.5	−59.5	−46.5	36% R. VIIIa, 16% R. VIIb, 1% R. VIIIb
5	2	0.05	8	−62	−28	8	−62	−27	3% R. VI, 2% Vermis VI, 1% R. VIIIa, 1% R. V

## Data Availability

All relevant data are within the manuscript. The research was performed under an IRB approval, and required the research subject to sign an informed consent. The in vivo data cannot be made publicly available, as this would violate Swedish law, since the research subjects did not agree to data sharing at the time of acquisition. According to Swedish law applicable to this study, the scope of the consent must be specific (Personal Data Act 1998:204; Swe. “Personuppgiftslagen”, http://rkrattsbaser.gov.se/sfst?bet=1998:204, accessed on 16 April 2021). Therefore, we are prohibited from sharing the data publicly for general. Data are available upon request from researchers who have ethical approval to Professor Pia Sundgren, at the Department of Clinical Sciences, Division of Radiology (Address: Diagnostic Radiology, Skåne University Hospital, Lund 22185, Sweden).
